# Lactate dehydrogenase elevations is associated with severity of COVID-19: a meta-analysis

**DOI:** 10.1186/s13054-020-03161-5

**Published:** 2020-07-24

**Authors:** Xiao-Yun Chen, Ming-Yao Huang, Zheng-wei Xiao, Sheng Yang, Xiang-Qi Chen

**Affiliations:** 1 Department of Respiratory Medicine, Fujian Medical University Union Hospital, Fuzhou, 350001 Fujian People’s Republic of China; 2 Department of General Surgery, The Fourth Affiliated Hospital, China Medical University, Shenyang, 110000 People’s Republic of China; 3 Department of Orthopaedics, Fujian Provincial Hospital, Fuzhou, 350001 Fujian People’s Republic of China

**Keywords:** COVID-19, Lactate dehydrogenase, Severe disease

Dear Editor,

The coronavirus disease 2019 (COVID-19) has become one of the most serious pandemics of the recent times. It is crucial and necessary to identify laboratory markers which could provide predictions for the severity as well as the prognosis of the disease in order to guarantee proper clinical care for the patients. Therefore, the aim of this meta-analysis is to explore the unclear association of lactate dehydrogenase (LDH) elevations and the severity of COVID-19.

We gained access to relevant literature by searching PubMed, EMBASE, and The Cochrane Library through June 3, 2020. We used the following terms: “Lactate Dehydrogenase,” “2019 novel coronavirus,” “2019-nCoV,” and “COVID-19”. Articles which provide data about patients with COVID-19 whether of severity or not (including either respiratory distress, ICU admission, and/or death) were selected [1]. LDH elevations were reported based on reference laboratory parameters for each study. Two investigators (X.Y. Chen, M.Y. Huang) independently extracted the data by discussing with the corresponding authors about any different opinions until they reach a mutual agreement. We applied the exact binomial method to extract the odds ratios (OR) in forest plots with 95% confidence interval (CI). The presumption of homogeneity was not accepted as a valid statement when *P* < 0.1 and *I*^2^ > 50%. All data were processed in the Stata version12.0 (StataCorp, College Station, TX).

After reviewing titles as well as abstracts, we continued to read the whole texts of the remaining articles. At last, a total of 6 articles were selected by this meta-analysis as shown in Table 1. Figure 1 summarizes the OR pooled by the selected studies, showing that elevated LDH values are related to an almost 12-times increase in the risks for severe COVID-19 with low heterogeneity being observed (OR, 12.43; 95% CI, 7.23–21.38; *P* < 0.001; *I*^2^, 8.2%). Therefore, the fixed-effects model was used for the meta-analysis.

**Table 1:** Characteristic of included studies (*n* no. of patients)

Study, year	Country	Females, *n* (%)	Median age	Total patients	Severe patients, *n* (%)	LDH (increased/total), *n* (%)	
						Severe	Non-severe
Huang, 2020	China	11 (26.8%)	49.0	41	13 (31.7%)	12/13 (92.3%)	17/27 (63.0%)
Wang, 2020	China	37 (54.0%)	42.0	69	14 (20.3%)	10/12 (83.3%)	15/49 (30.6%)
Zhou, 2020	China	72 (38.0%)	56.0	191	54 (28.3%)	53/54 (98.1%)	70/130 (53.8%)
Zhang, 2020	China	66 (57.4%)	49.5	115	31 (30.0%)	17/31 (54.8%)	9/84 (10.7%)
Wan, 2020	China	63 (46.7%)	47.0	135	40 (29.6%)	30/40 (75.0%)	28/95 (29.5%)
Chen, 2020	China	4 (19.0%)	56.0	21	11 (52.4%)	10/11 (90.9%)	1/10 (10.0%)

**Fig. 1:**
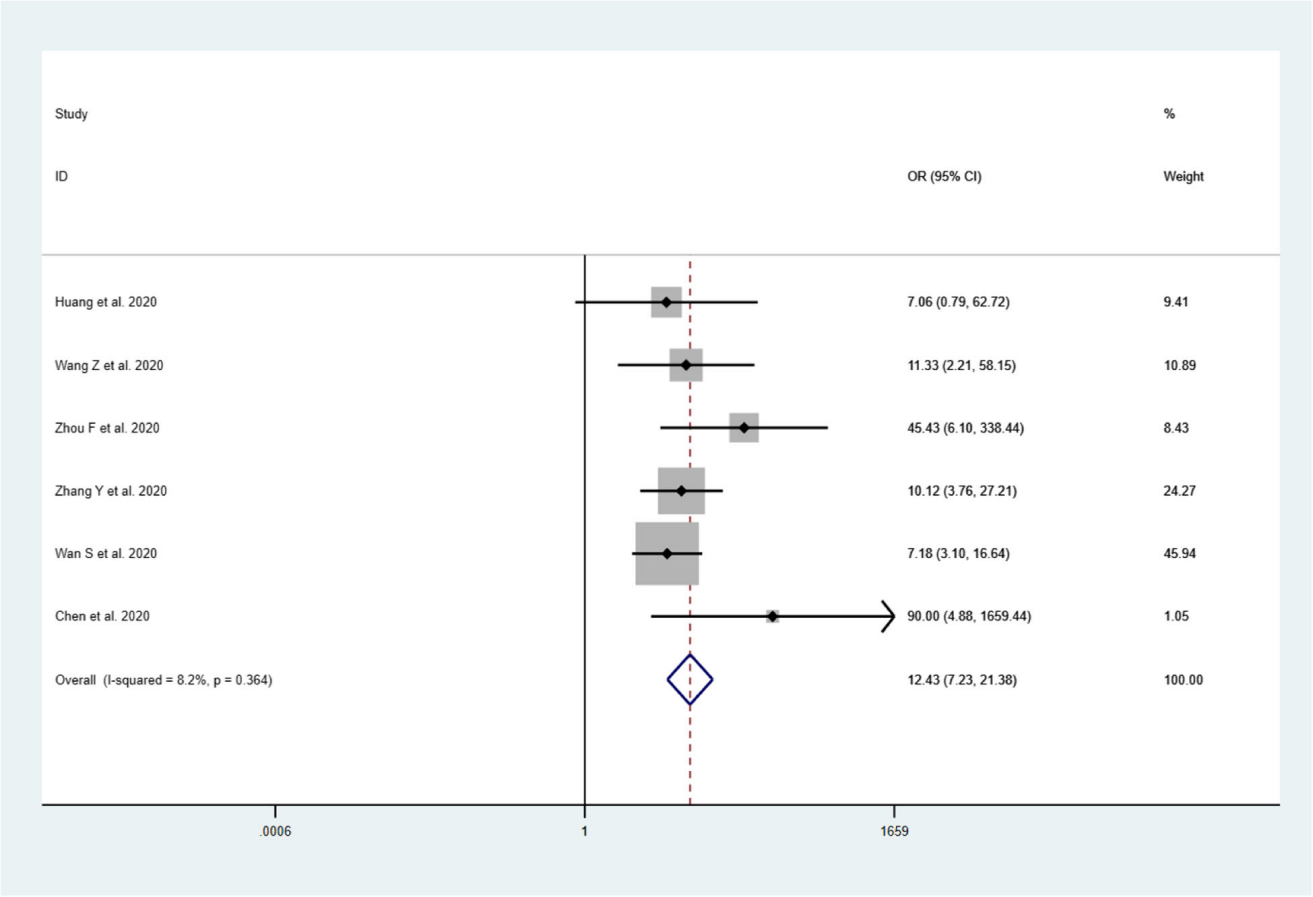
OR and 95% CI of LDH values beyond normal reference range for the prediction of severe COVID-19

Findings of this meta-analysis suggest that measuring LDH might be helpful in predicting whether COVID-19 will evolve into a more severe state. This conclusion has its reasonable grounds. LDH, a cytoplasmatic enzyme, can be found in basically every main organ system. If cell lysis occurs, or cell membranes are damaged, LDH will be released into the extracellular space [2]. In acute inflammation, the changes in cells are manifested as neutrophils influx which may be the cause of the lung damages occurred and the production of the toxicity of cells [3–5]. The cytotoxicity of neutrophils was suggested as being related to ARDS [6]. LDH elevations are related to the aforementioned cell damage or inflammation or both.

Our meta-analysis has a few limitations. All studies are from China. In order to exclude the influence of the genetic factor, data from other countries should be compared. Due to the nature of reporting in the emerging outbreak, we do not conduct a risk of bias assessment. Future analyses need to aim at confirming the results of this paper as well as pooling data to find other laboratory markers of severe COVID-19.

We confirm that LDH elevations are associated with increased risk for severe COVID-19. It can be used to provide predictions for the severe disease during hospitalization to guarantee proper clinical care for the patients.

## Data Availability

The datasets used and/or analyzed during the current study are available from the corresponding author on reasonable request.

## Abbreviations

LDH: Lactate dehydrogenase
COVID-19: Coronavirus disease 2019
ICU: Intensive care unit
OR: Odds ratio
CI: Confidence interval
*n*: No. of patients
ARDS: Adult respiratory distress syndrome
